# Patterns and drivers of the belowground bud bank in alpine grasslands on the Qinghai-Tibet Plateau

**DOI:** 10.3389/fpls.2022.1095864

**Published:** 2023-01-18

**Authors:** Wencheng Li, Aiping Huang, Tiancai Zhou, Miao Liu, Sujie Ma, Ningning Zhao, Xiangtao Wang, Jian Sun

**Affiliations:** ^1^ Key Laboratory of Alpine Vegetation Ecological Security, Tibet Agriculture and Animal Husbandry University, Nyingchi, China; ^2^ State Key Laboratory of Tibetan Plateau Earth System, Environment and Resources (TPESER), Institute of Tibetan Plateau Research, Chinese Academy of Sciences, Beijing, China; ^3^ College of Resources and Environment, Tibet Agriculture and Animal Husbandry University, Nyingchi, China; ^4^ Qiangtang Alpine Grassland Ecosystem Research Station (jointly built with Lanzhou University), Tibet Agricultural and Animal Husbandry University, Nyingchi, China; ^5^ College of Grassland Science and Technology, China Agricultural University, Beijing, China; ^6^ College of Animal Science, Tibet Agricultural and Animal Husbandry University, Nyingchi, China

**Keywords:** alpine grasslands, Qinghai-Tibet Plateau, bud bank, vegetation reproduction, ecological restoration, clonal plants

## Abstract

**Introduction:**

In grassland ecosystems dominated by asexual plants, the maintenance, renewal, and resistance of plant populations to disturbance are more dependent on the belowground bud bank (BBB). However, the response of the BBB to environmental factors in the alpine grassland of the Qinghai-Tibet Plateau (QTP) is still unknown.

**Methods:**

Therefore, a transect survey was conducted to measure the size and scale of BBB and 21 factors in the alpine grassland of the QTP. In addition, the critical driving factors of BBB were screened by boost regression tree analysis, and a structural equation model (SEM) was employed to express the path coefficients of the key factors on the BBB size.

**Results:**

The results showed that BBB size had no significant geographical pattern in the QTP, and the BBB size was mainly accounted for by soil leucine aminopeptidase (LAP, 17.32%), followed by Margalef and Shannon -Wiener indices of plants (12.63% and 9.24%, respectively), and precipitation (9.23%). SEM further indicated significant positive effects of plant diversity (scored at 0.296) and precipitation (scored at 0.180) on BBB size, and a significant negative effect of LAP (scored at 0.280) on BBB size.

**Discussion:**

Generally, the findings allow for better understanding of the regulated mechanisms of BBB size and the importance of the role of bud bank in the restoration of the grassland ecosystem.

## 1 Introduction

Grasslands are one of the major terrestrial ecosystem types, covering approximately 37% of global land area (O'Mara, 2012), and they play a crucial role in energy and material cycles, global climate change, and global carbon balance (Scurlock et al., 2002). Alpine grasslands have the largest distribution area of plant types, covering more than 50% of the Qinghai-Tibet Plateau (QTP) (Liu et al., 2017). Since the ecological structure and function of alpine grasslands are sensitive to global changes, they can serve as a warning sign for the ecological environment (Sun et al., 2020). Furthermore, the clonal nature of most herbaceous plants allows them to play an important role in maintaining the stability of grassland ecosystems, due to their ability to reproduce both sexually and asexually to complete renewal iterations (Lee, 2004). The storage status of the seed and bud bank is particularly important as they are two sources of vegetation renewal. Some studies have shown that annual plants mainly rely on seed production to produce offspring; however, in grassland ecosystems dominated by perennial grasses, plant population maintenance and regeneration, community composition and dynamics, and responses to environmental changes and anthropogenic disturbances are more dependent on the belowground bud bank (BBB) (Deng et al., 2010; Teniwu et al., 2021).

Additionally, the BBB is highly resistant to various disturbances. For example, previous studies found that in North American tallgrass prairie, 99% of vegetation regeneration after fire treatment was dependent on the bud bank (Hartnett et al., 2006). Similarly, in the Hulunbuir meadow grassland of northeast Inner Mongolia, BBB plays an important role in resisting extreme drought (Teniwu et al., 2021). Generally, grasslands with large BBB sizes are likely to be more responsive to future climate change or other phenomena (i.e., nutrient enrichment), and similarly, have greater resistance to phenomena such as invasive alien species (Carter and Vanderweide, 2014). Other studies have also demonstrated that the potential population productivity of grasslands is determined by the total number of bud banks and the number of active buds (Ott and Hartnett, 2012). Environmental changes that deplete bud banks or prevent their formation may lead to the loss of vegetation resilience and plant species diversity (Ott et al., 2019), and changes in these environmental factors may affect the input and output of bud banks. Although the maintenance, renewal, and resistance to disturbance of plant populations in grassland ecosystems with asexual plants are more dependent on the BBB, the response of the BBB to various environmental factors in alpine grasslands of the QTP is still unknown.

A transect survey was conducted to measure the bud bank size and 21 environmental factors, and a structural equation model (SEM) was then designed to investigate the key factors that affect the BBB on the QTP. Specifically, the following questions were addressed: (i) What are the size and geographical spatial distribution patterns of the BBB in the QTP? (ii) What are the key factors affecting the BBB in the QTP? This study was aimed to further understand and predict the plant community dynamics of alpine grasslands, as well as the input and output trends of BBB size in the alpine grasslands of the QTP.

## 2 Materials and methods

### 2.1 Study area and sampling

The QTP, which is the largest geomorphological unit on the Eurasian continent, has an area of approximately 2.5 million km^2^, with 60% of the land covered by alpine grassland (Hong et al., 2016; Zhou et al., 2017). It is also one of the terrestrial ecosystems most sensitive and vulnerable to climate change (Klein et al., 2007).

**Figure 1:**
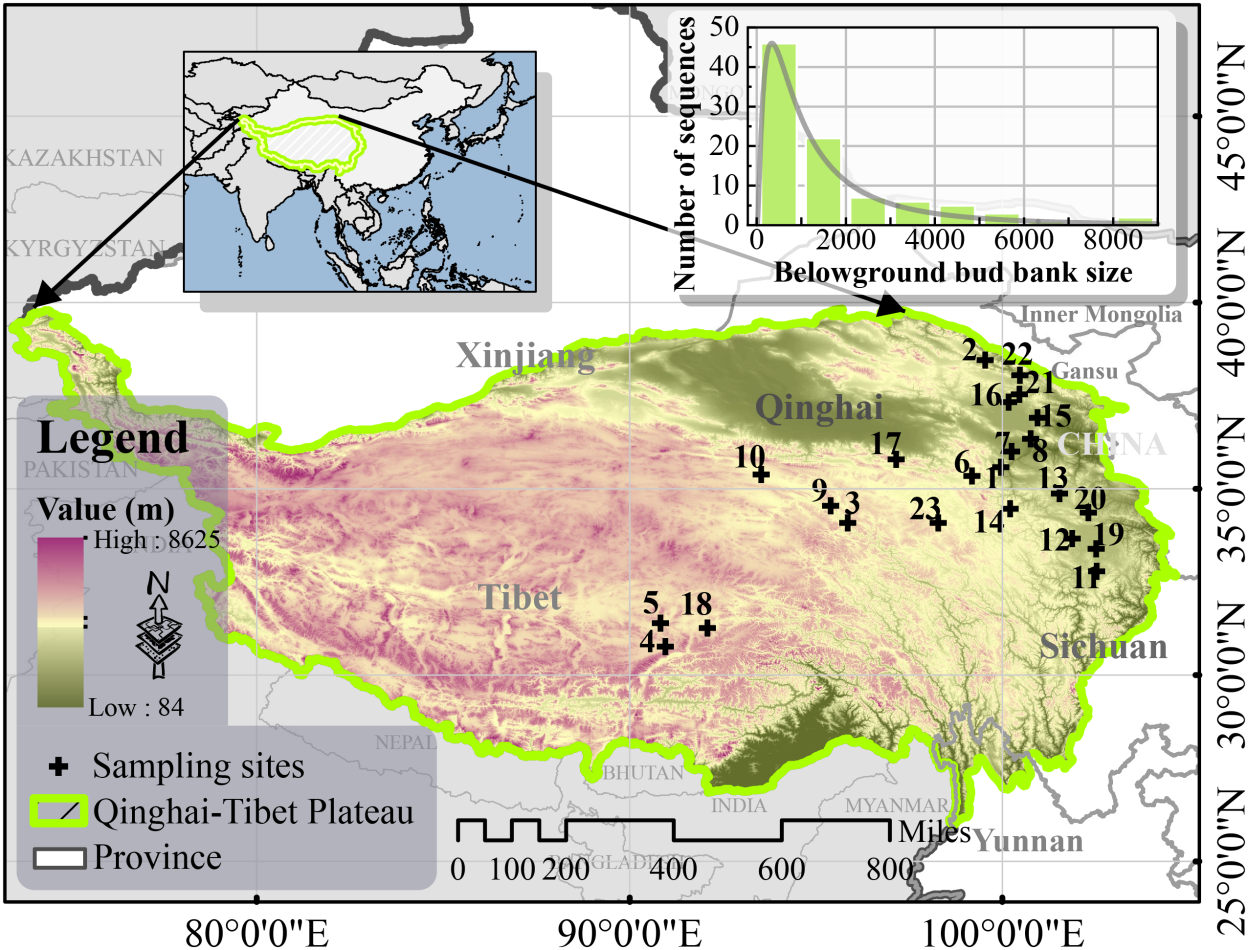
Sample sites in the study area (Built-in frequency distribution histogram of belowground bud bank size).

The study sites were located around the northeastern hinterlands of the QTP. The bud library data had four replicates, while all the other sampled data had three replicates. BBBs, plant communities, and soil were sampled from 23 study sites (90-103°E, 30-39°N) in the northeastern and central parts of the QTP, including the northwest region of Sichuan Province, Qinghai Province, and the Tibetan Autonomous Region, China (**Figure 1**), during the peak growing season (July-August) of 2022.

The grassland transects included alpine meadows and alpine steppes at altitudes between 3023 m and 4743 m. The study site annual temperatures ranged from −4.34 to 3.46°C, while annual mean precipitation ranged between 209.89 mm and 861.59 mm, additionally, the precipitation and temperature on the QTP decreased from the southeast to the northwest (Sun et al., 2020). Details of the dominant species and the geographic information of the sample sites are shown in **Table 1**.

**Table 1:** Spatial information of sample sites and dominant species of plots.

Sites	Coordinates	Altitude (m)	Dominant species
1	99°57′57.60″ E 35°35′45.60″ N	3281.00	*Carex tristachya, Carex myosuroides, Leymus chinensis*
2	99°32′16.80″ E 38°27′32.40″ N	3239.42	*Carex parvula, Carex myosuroides*
3	95°51′32.40″ E 34°05′34.80″ N	4223.91	*Stipa purpurea, Carex myosuroides*
4	90°57′50.40″ E 30°46′22.80″ N	4742.97	*Saussurea arenaria, Stipa purpurea*
5	90°50′02.40″ E 31°24′07.20″ N	4545.23	*Stipa purpurea*
6	99°11′12.55″ E 35°21′39.17″ N	4158.00	*Leontopodium pusillum*
7	100°14′33.79″ E 36°00′37.41″ N	3118.00	*Stipa purpurea, Poa tibetica Munro*
8	100°45′23.49″ E 36°21′10.13″ N	3186.00	*Stipa purpurea, Poa tibetica Munro*
9	95°23′20.06″ E 34°32′56.05″ N	4219.00	*Stipa purpurea, Artemisia hedinii*
10	93°31′35.30″ E 35°23′46.45″ N	4459.00	*Polygonum viviparum, Carex alatauensis, Oxytropis ochrocephala*
11	102°32′13.20″ E 32°47′45.60″ N	3426.50	*potentilla bifurca*
12	101°52′12.00″ E 33°40′22.80″ N	3560.00	*Carex alatauensis*
13	101°32′06.76″ E 34°52′56.03″ N	3575.60	*potentilla bifurca, Stipa purpurea, Oxytropis ochrocephala*
14	100°12′10.80″ E 34°28′48.00″ N	3715.00	*Stipa purpurea, Ligularia sibirica, Poa tibetica*
15	100°56′16.80″ E 36°55′04.80″ N	3050.67	*Stipa purpurea*
16	100°09′46.80″ E 37°20′20.40″ N	3313.54	*Carex tristachya, Carex myosuroides*
17	97°53′27.24″ E 35°17′28.00″ N	4604.00	*Poa tibetica Munro*
18	92°05′42.00″ E 31°16′15.60″ N	4461.48	*Stipa purpurea, Carex myosuroides*
19	102°31′03.30″ E 33°23′58.68″ N	3475.00	*Stipa purpurea, Poa tibetica*
20	102°18′16.36″ E 34°22′03.68″ N	3480.00	*Anaphalis xylorhiza, Poa tibetica*
21	100°27′04.77″ E 37°32′21.43″ N	3559.00	*Lancea tibetica, Artemisia hedinii, Elymus mutans*
22	100°28′39.67″ E 38°03′38.45″ N	3023.00	*Carex tristachya, Stipa purpurea*
23	98°17′03.84″ E 34°51′23.44″ N	4221.00	*Carex tristachya*

### 2.2 Climate data measurement

It is clear that these climate data were predictive. For each meteorological station, the annual mean temperature (AMT) and annual mean precipitation (AMP) from the Meteorological Information Center of the Chinese National Bureau of Meteorology (http://data.cma.cn/) were compiled using Excel (Microsoft Excel 2016). Data were then interpolated by Anusplin 4.2 (Centre for Resource and Environmental Studies, Australian National University, Canberra) to obtain the grid data of climate for the study area, and these sample site data were extracted with ArcGIS 10.2 (ESRI, Inc., Redlands, CA, USA).

### 2.3 Soil data measurement

Soil samples were measured between 0 and 30 cm for the following soil physiochemical data: soil available phosphorus content (SAP) measured by the Olsen method (Zhou et al., 2020), soil ammonium nitrogen (NH_4_^+^-N) and soil nitrate nitrogen (NO_3_^−^-N) by Elementar TOC analyzer (Liqui TOCC II, Germany) (Hu et al., 2020), soil total carbon content (TC) and soil total nitrogen content (TN) by MACRO cube elemental analyzer (Elementar Analysensysteme GmbH, Germany) (Zhou et al., 2020), soil pH (pH) and soil temperature (ST) by portable time domain reflectometer (TDR 100, Spectrum Technologies Inc., Chicago, IL, USA), and soil moisture content (SMC) by drying (Jiang, 2019).

Soil enzyme activity, a microplate fluorometric assay, and a soil enzyme assay kit were used to determine the activity of soil β-glucosidase (βG, EC:3.2.1.21), soil leucine aminopeptidase (LAP, EC:3.4.11.1), soil polyphenol oxidase (PPO; EC:1.14.18.1), soil alkaline phosphatase (AKP, EC:3.1.3.1), and soil acid phosphatase (ACP, EC:3.1.3.2). Their enzymatic activity was determined by the degree to which the enzyme catalysed the reaction of the corresponding substrate. AKP and ACP were analyzed using phenyl disodium phosphate as a substrate (βG with p-nitrophenyl β-D-glucopyranoside, LAP with L-leucine-p-nitroanilide, and PPO with pyrogallol). For βG, the production of 1 μmol of p-nitrophenol per g of soil sample per day was defined as one unit of enzyme activity. For PPO, the production of 1 mg of purple gallic substance per g of soil sample per day was defined as one unit of enzyme activity. For soil LAP enzyme, the production of 1 nmol of p-nitroaniline per g of soil sample per minute was defined as one unit of enzyme activity.

For AKP and ACP at 37 °C, 1 nmol of phenol per g of soil released per day was one unit of enzyme activity; notably, AKP was measured in an alkaline environment and ACP in an acidic environment.

### 2.4 Plant data measurement

Three replicate plots (50 cm×50 cm) were randomly selected from each study site (10 m×10 m) to obtain plant samples for the measurement of each plant indicator.

The number of species of above-ground plants in the plot were counted and the relative abundance of each calculated. Based on this, the following plant community species diversity indicators were obtained: Margalef richness index (*R*, Eq.1), Shannon-Wiener index (*H’*, Eq.2), Pielou’s evenness index (*J’*, Eq.3), and Simpson index (*D*, Eq.4).


(1) $$R=\frac{S-1}{\ln N}$$

(2) $$H^{\prime }=-\sum_{i=1}^{s}P_{i}\ln P_{i}$$

(3) $$J^{\prime }=\frac{H}{\ln S}$$

(4) $$D=\sum_{i=1}^{s}P_{i}^{2}$$

where *S* is the number of species in the sample, *N* is the total number of each plant in the sample, and *P_i_* is the relative abundance of the *i* th species in the community (Smith and Wilson, 1996).

Additionally, above- and below-ground samples were oven-dried at 65°C to constant mass, then get aboveground biomass (AGB) and belowground biomass (BGB) by weighing.

### 2.5 Belowground bud bank data

BBB is a collection of dormant meristems below the ground and on some surfaces that are available for vegetative reproduction. BBB size was calculated using soil cores (Qian et al., 2017). To maintain the natural connection between plants above and below ground, all individuals from each plot were excavated to identify the category of BBBs. The soil core was then completely removed by shaking out the soil block out or soaking the corer in water, and from which then had buds counted. The most visible buds were directly selected and counted, while other indistinguishable buds were dissected before being counted. Only soil-borne buds were considered in this study, and not those at the surface.

### 2.6 Statistical analysis

**Figure 2:**
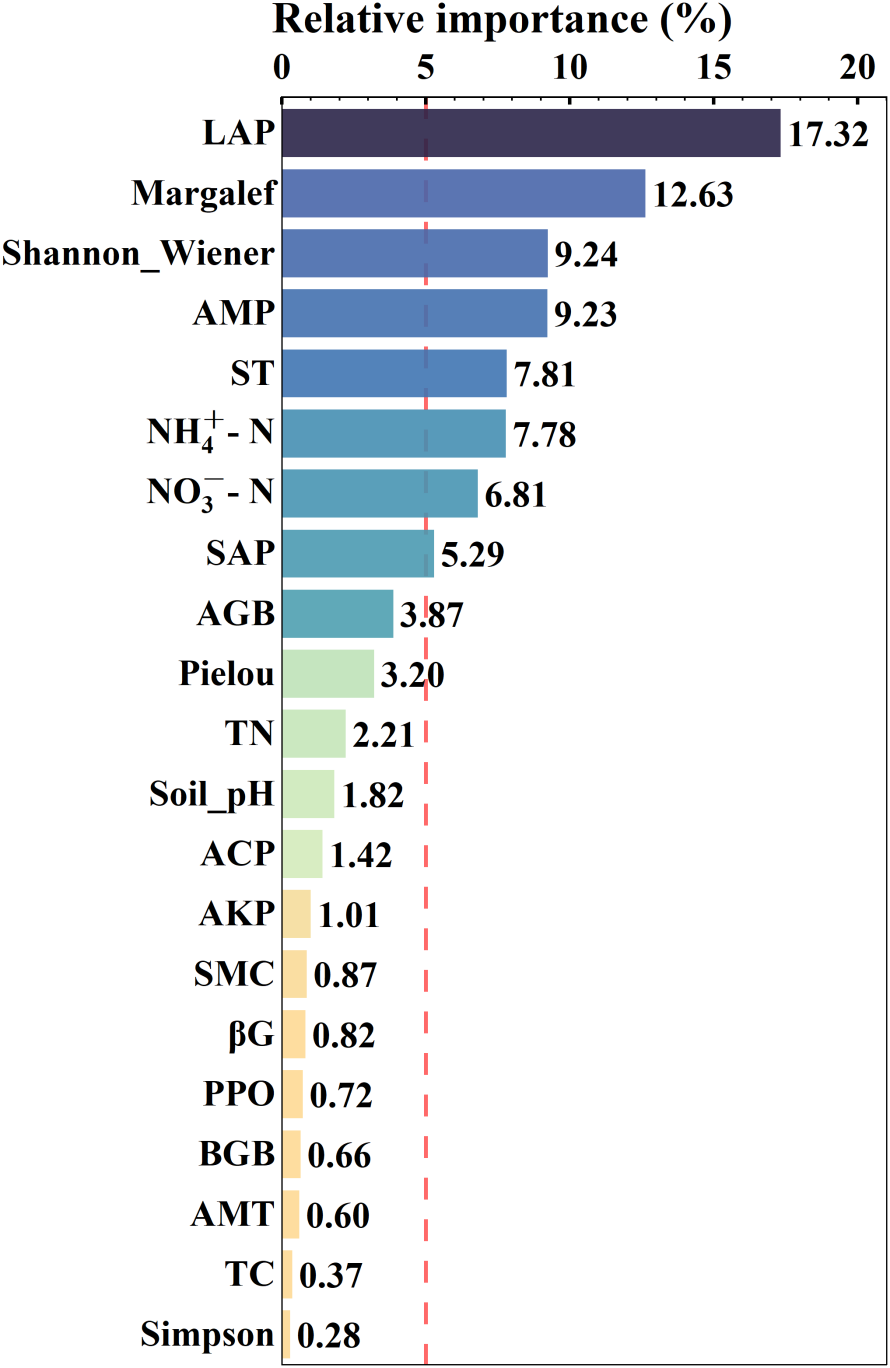
Results of boost regression tree analysis, screening factors by the dashed vertical red line (i.e., greater than 5%), the darker the color, the greater the relative importance. The environmental factors from top to bottom are LAP (soil leucine aminopeptidase, EC:3.4.11.1), Margalef (Margalef index), Shannon_Wiener (Shannon-Wiener index), AMP (annual mean precipitation), ST (soil temperature), NH_4_^+^-N (soil ammonium nitrogen), NO_3_^−^-N (soil nitrate nitrogen), SAP (soil available phosphorus content), AGB (aboveground biomass), Pielou (Pielou’s species evenness index), TN (soil total nitrogen content), Soil_pH, ACP (soil acid phosphatase, EC:3.1.3.2), AKP (soil alkaline phosphatase, EC:3.1.3.1), SMC (soil moisture content), βG (soil β-glucosidase, EC:3.2.1.21), PPO (soil polyphenol oxidase, EC:1.14.18.1), BGB (belowground biomass), AMT (annual mean temperature), TC (soil total carbon content), Simpson (Simpson’s diversity index).

Excel and Origin 2022 (Origin Lab Corporation, Northampton, MA, USA) were used to process the experimental data and make simple charts. To determine the important driving factors of BBB size, a boost regression tree (BRT) analysis was then used with the “*gbm*” package of R (version 4.2.1; R Development Core Team) (**Figure 2**). Then, according to the above BRT analysis, factors with >5% relative importance were screened to explore the relationship between greater importance factors (LAP, Margalef, Shannon -Wiener, AMP, ST, NH_4_^+^-N, NO_3_^−^-N, SAP) and BBB size. To divide the factors with the same category into a group (containing two or more factors), principal component analysis (PCA) was conducted with the packages “*FactoMineR*”, “*factoextra*”, and “*corrplot*” in R, and PCA was used to transform multiple factor variables into a set of variables (Zhou et al., 2021) (**Supplementary Figure 1**). Ultimately, to reveal the mechanisms by which environmental factors influence BBB size, an analysis using path coefficients was undertaken in Amos software (17.0.2, IBM SPSS Inc.), which includes a synthesis of factor analysis, path analysis, and maximum likelihood analysis (Grace, 2002; Doncaster, 2007). Notably, 39.9% and 75.6% of the total variance for the groups were explained by the first component (PC1), which was then introduced as a new variable in the SEM.

## 3 Results

### 3.1 Geography pattern of BBB on the QTP

**Figure 3:**
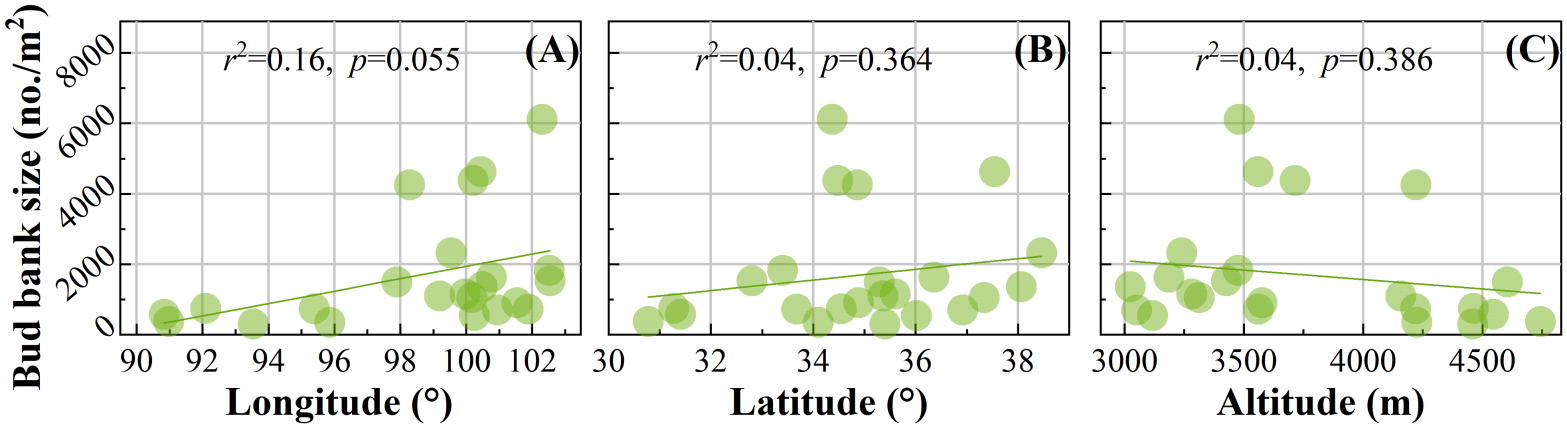
Spatial pattern of belowground bud bank. The relationships between BBB and **(A)** longitude, **(B)** latitude, and **(C)** altitude.

Through linear fitting of the scatterplot, no significant difference was found between BBB size and geographical pattern (**Figure 3**). However, in terms of fit line trend, the BBB size tends to increase with increasing latitude and longitude (**Figures 3A, B**) and tends to decrease with increasing altitude (**Figure 3C**).

### 3.2 The relative importance of the environmental factors to BBB size

The results of BRT analysis demonstrated the relative importance of all 21 predictors of BBB size (**Figure 2**). The highest factor influencing BBB size was enzyme LAP (17.32%), followed by, in descending order of relative importance, plant diversity indices Margalef (12.63%), Shannon -Wiener (9.24%), climate AMP (9.23%), soil ST (7.81%), NH_4_^+^-N (7.78%), NO_3_^−^-N (6.81%), and SAP (5.29%). The other factors were less than 5% and not included in the discussion.

### 3.3 Driving mechanism of key factors on BBB size

**Figure 4:**
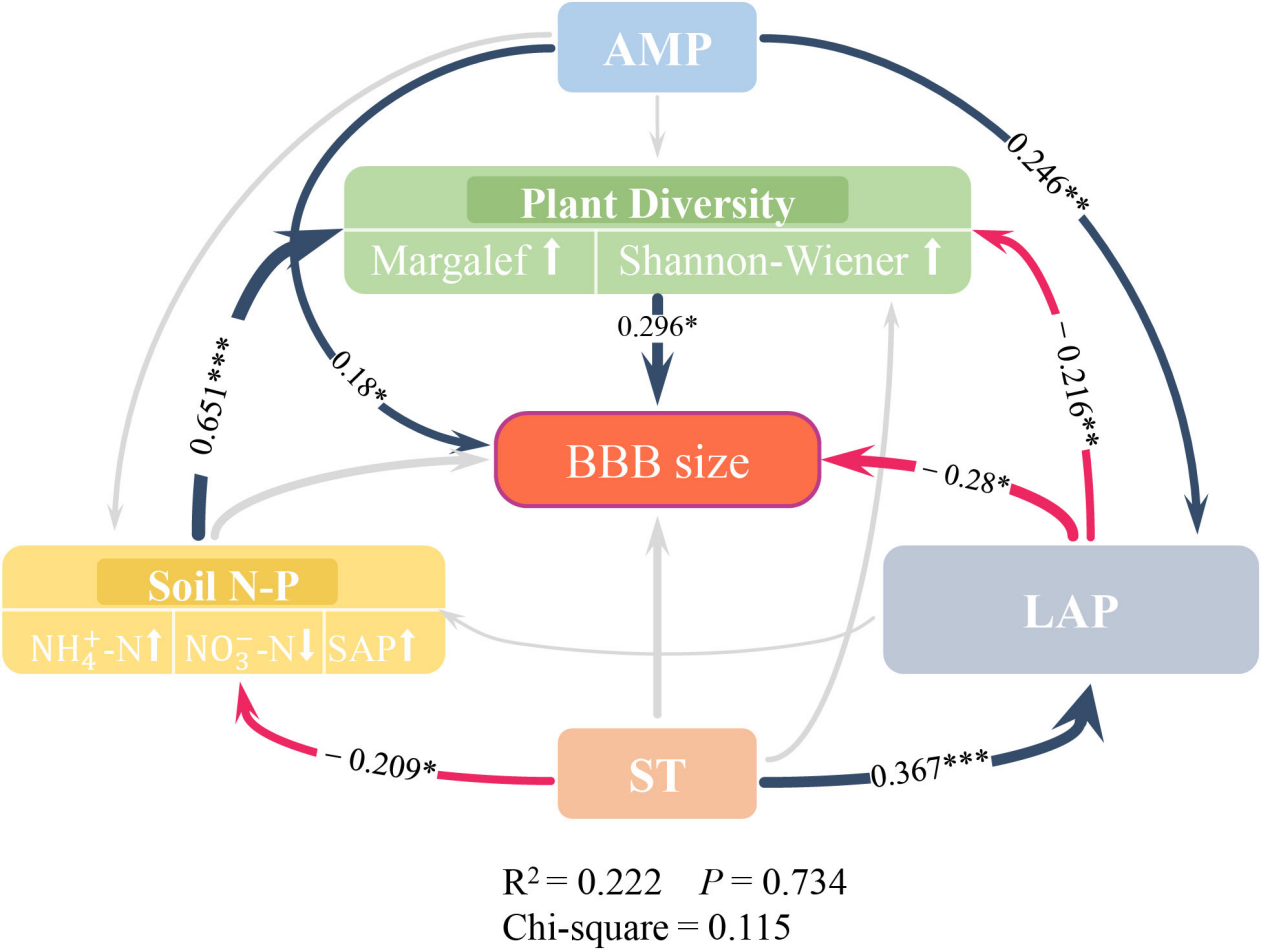
Structural Equation Modeling of belowground bud bank and environmental factors. Direct and indirect impacts of key factors on belowground bud bank size. The colored arrows and gray arrows represent significant and non-significant relationships, respectively (* *p*<0.05, ** *p*<0.01 and *** *p*<0.001, only the values of path coefficients with significance were presented in SEM); Positive correlations are displayed in dark blue arrows, and negative correlations are displayed in red arrows; The arrows thickness represent the values of path coefficients. The thicker the path, the bigger the value. Of note, the variables in the double-layer rectangles represent the screened factors (results of BRT analysis in **Figure 2**) that were used in PCA, and arrows pointing up or down in double-layer rectangles represent the positive or negative relationships between the observed variables and potential variables. The environmental factors from top to bottom are AMP (annual mean precipitation), Plant Diversity (Margalef [Margalef index], Shannon_Wiener [Shannon-Wiener index]), BBB (belowground bud bank), Soil N-P (NH_4_^+^-N [soil ammonium nitrogen], NO_3_^−^-N [soil nitrate nitrogen], SAP [soil available phosphorus content]), LAP (soil leucine aminopeptidase, EC:3.4.11.1), ST (soil temperature).

SEM indicated that AMP, soil LAP enzymes, and “Plant Diversity” play a key driving role in BBB size (**Figure 4**). In this model, both AMP (standardized effect = 0.18) and plant diversity (0.296) had significant direct positive effects on BBB size, and LAP (−0.28) had a significant negative effect on BBB size.

Although soil N-P had no direct effect on BBB size (*p*>0.05), it had a significant indirect effect through the positive effects of plant diversity on BBB size (standardized effect = 0.193). Although ST had no direct effect on BBB size (*p*>0.05), it had an indirect effect on BBB size through LAP (−0.103) and also on BBB size through soil N-P and plant diversity (−0.04). In addition, ST had a significant positive effect on LAP (0.367) and a significant negative effect on soil N-P (−0.209), AMP had a significant positive effect on LAP (0.246), and LAP had a significant negative effect on plant diversity (−0.216).

## 4 Discussion

In this study, the BBBs size in the alpine grasslands on the QTP was approximately within the range of 312 - 6120 buds per m^2^ (**Figure 1**; **Supplementary Table 1**), with a mean value ranging from 1690.43 buds per m^2^ to 6000 buds per m^2^ which is slightly larger than that of the loess hilly -gully region in a soil erosion environment, which was reported by Du et al. (2013) as 600-1300 buds per m^2^. Similarly, the BBB size range in tallgrass prairie in the Flint Hill region of the United States was similar to that of the QTP, ranging from 600-1800 buds per m^2^ (Benson et al., 2004). In south-central Nebraska, the BBB size in restored grassland was higher than that in the QTP, which is approximately 300-3500 buds per m^2^ (Carter et al., 2012). However, BBB size also varied considerably among grassland types within the same North American prairies, with a desert grassland values much smaller than those in this study (146 buds per m^2^) and tallgrass prairie value slightly higher (2450 buds per m^2^) (Dalgleish and Hartnett, 2006). It is common to use the number of buds per unit area to determine BBB size; however, some studies have used “per tiller” or “per volume” (Peterson, 1979; Baret et al., 2004; Ithurrart et al., 2019; Russell et al., 2019). There are certain differences in BBB size in different ecosystems, and the size varies depending, to a limited extent, on the researchers’ methods and the time of the investigation (Zhao et al., 2015).

This study shows that BBB has no significant relationship with latitude, longitude, and altitude on the QTP (*p*>0.05) (**Figure 3**). However, in the European Alps, the BBB size of plant communities tends to increase with altitude (Evette et al., 2009). The adaptation of the alpine plants on the QTP to survive in both high and low altitude climatic conditions has possibly resulted in little size difference. In comparison, other studies have shown that all bud bank traits did not significantly correlate with geographical location (longitude, latitude, and elevation) (Wang et al., 2021), which is consistent with this study.

Each factor and the process of BBB size are a result of multiple interactions, and variations exist between ecosystems and populations (Zhao et al., 2015). In this study, AMP had a significant positive effect on BBB size, which is consistent with results found elsewhere (Dalgleish and Hartnett, 2006; Qian et al., 2017; Teniwu et al., 2021). Rainfall directly affects the inputs and outputs of the BBB. Under high rainfall, the input to the bud bank will increase, and under drought conditions, the bud bank will reflect the Bet-Hedging strategy (Philippi, 1993), i.e., some buds participate in sprouting, while others remain dormant in response to unknown disturbances. Of note, there was a lag in the response of BBB size to AMP at sites with less AMP (200-300 mm), and it has been found that BBB size could only be maintained at a similar level to the previous year’s BBB size, even with higher rainfall in the current year (Zhang, 2009). In addition, in North American tallgrass prairie there are certain thresholds for the responses of BBB size to environmental changes, with the size remaining largely stable, while aboveground community richness reduces when precipitation is reduced by 80% (Vanderweide and Hartnett, 2015). In addition, drought causes a 30-40% reduction in aboveground net primary productivity in North American tallgrass prairie, but has no significant effect on BBB size (Hoover et al., 2014). This also implies that the BBB plays an important role in resisting and recovering from extreme environments. At the regional scale, the response of BBB size to drought is inconclusive (Teniwu et al., 2021), increasing (Chen et al., 2015), decreasing (Dalgleish and Hartnett, 2006), or stable (Carter et al., 2012). In this study, the data showed that BBB size increased linearly with soil moisture content, which may be caused by differences in grassland vegetation types and plant communities. Additionally, previous studies have shown that water controls the distribution and composition of the BBB in plant communities, which can provide important information for predicting the dynamics of plant communities in alpine meadows (Ding et al., 2019). Overall, on the QTP, the BBB size followed a rainfall gradient with a decreasing trend toward drier and warmer areas.

Precipitation not only affects BBB but also influences the development of alpine grassland vegetation to a large extent (Sun et al., 2020). Other studies have shown that the response of vegetation to environmental change may be mediated through bud bank dynamics (Ott et al., 2019). The size and composition of BBB are greatly dependent on the aboveground vegetation organs to which buds are attached, in contrast to the soil seed bank (Klimešová and Klimeš, 2007). In general, the existence of belowground buds is dependent on the bearing organs of the plant ontology, surviving with the parent plant, with some variation introduced by habitat inconsistency. However, habitat inconsistencies often lead to differences and similarities in biodiversity (Kreft and Jetz, 2007). Plant diversity has a significant positive effect on BBB size. It has been shown that at the population level, total BBB size increases significantly with increasing population density, but for a certain species, only moderate population density significantly increases the BBB size of populations and individuals dominated by that species (Zhang et al., 2019). Furthermore, other work has shown that several sampled plots in alpine meadows with the highest plant diversity have the largest BBB (Ding et al., 2019). Most studies on plant diversity and bud banks have imposed anthropogenic disturbances to determine the critical role of BBB restoration in grassland ecosystems (Vanderweide and Hartnett, 2015; Hiiesalu et al., 2021). Overall, aboveground plant diversity will decline or possibly be lost with interference, but after disturbance, the BBB plays an important role in the recovery and renewal of vegetation. The increase in BBB size is believed to be related to interspecific competition; in general, the richer the diversity, the more intense the competition, and individual plants will clone themselves quickly (via asexual reproduction and bud bank) to enable utilization of resources, which in turn leads to an increase in BBB.

Finally, soil acts as a substrate for grass plants to survive, and the soil nutrients and contents have critical impacts on BBB size. Among them, soil nutrition is an important factor influencing the completion of the bud bank, and in general, BBB size increases with an increase in the nutrient substrate (Mcintyre and Raju, 1967; Klimeš and Klimešová, 1999). Therefore, soil nutrients are beneficial for the establishment and input of BBB. In this study it was found that soil N-P and soil LAP enzymes had indirect and direct significant negative effects on BBB size, respectively (**Figure 4**). It has been found that soil nitrogen (N) may be an inducing factor for bud dormancy or germination (Tomlinson and O'Connor, 2004). Under a low N level, the competition between the bud and leaf system for N also leads to the limitation of bud growth (Peterson, 1979), thus reducing bud input.However, Williamson et al. (2012) found a low N level may stimulate bud sprouting, resulting in the output of young buds, thus increasing BBB size. In other studies, BBB size was positively correlated with soil available nitrogen (SAN, the sum of NH_4_^+^-N and NO_3_^−^-N) (Wilson et al., 2016; Wang et al., 2021) and negatively correlated with SAP, while an increased soil N/P ratio had negative effects on plant diversity (Wilson et al., 2016). There are some differences between such a conclusion and this study’s results (**Figure 4**), the reseaon being that soil LAP enzyme mediates the process of available nitrogen conversion, and that other studies have shown soil LAP enzyme activity reflects the supply capacity of SAN (Burns et al., 2013). That is, the higher the enzyme activity, the greater the conversion of TN to SAN, which indirectly affects the BBB. However, this study’s results showed that the LAP enzyme was not correlated with SAN and was significantly negatively correlated with BBB size (**Figure 4**). The mechanisms involved are unclear and little research on this has been conducted. Some studies have found that N application has adverse effects on sexual reproduction (mainly the seed bank) and favorable effects on clonal reproduction (mainly the bud bank) (Li et al., 2021). These results indicated that N addition had a positive effect on the clonal propagation of perennial grass and could also improve the dominance of bud banks in the growth and reproduction of grassland, whereas there was uncertainty with phosphorus addition. In summary, under the conditions of nitrogen and phosphorus dominance, the joint effect of the two on the bud bank will be nitrogen-dominated and have a positive effect. It may be possible to undertake research on the effect of phosphorus addition on the BBB in the future, in order to fill the gap in the understanding of this.

## 5 Conclusion

In conclusion, by linking BBB size to influencing factors, such as geography, climate, soil, and plants at the community level of the QTP, this study comprehensively described the interaction process of different factors affecting BBB size. First, it was found that BBB size in the alpine grasslands of the QTP has no geographical pattern. Second, BBB size shows a higher sensitivity response to plant diversity, soil N-P, and soil LAP enzyme factors. Finally, BBB size was positively and significantly correlated with AMP and plant diversity, and significantly correlated negatively with the soil LAP enzyme. The results provide some background support for subsequent studies of BBB on the QTP and indicate that bud bank dynamics could predict future plant community dynamics to a certain extent. Consequently, the process of belowground asexual reproduction in grassland ecosystems deserves extensive attention, as ecological restoration and renewal of grasslands mainly depend on the bud bank.

## Data availability statement

The original contributions presented in the study are included in the article/**Supplementary Material**. Further inquiries can be directed to the corresponding author.

## Author contributions

WL wrote the manuscript and executed the technical assays and statistical analysis. WL, JS and XW designed the experiment. WL, AH, ML, SM, NZ, JS collected and analysed the data. WL, AH, NZ drew the graphs. JS and TZ reviewed and revised the manuscript. All authors contributed to the article and approved the submitted version.
