# Supplementary material for: Patterns and drivers of the belowground bud bank in alpine grasslands on the Qinghai-Tibet Plateau
Source: Front Plant Sci. 2023 Jan 18;13:1095864. doi: 10.3389/fpls.2022.1095864 (PMC9893863; doi:10.3389/fpls.2022.1095864)
Supplement: Supplementary file 1 [file DataSheet_1.docx]

Supplementary Material

# Supplement Tables:

**Supplement Table 1** Descriptive statistics and normality test results of each index of the sample points. And *p*>0.05 means that the data were normally distributed, and *p*<0.05 means the opposite. The “Mean”, “SE”, “Max”, “Min”, “Mid” and “Quartiles” are the average, standard error, maximum, minimum, median and “lower quartiles, upper quartiles” values, respectively.

| Survey indicator | *P*  （Shapiro–Wilk test） | Mean ± *SE* | | | | Max | Min | Mid (Quartiles) | | | | | |
| --- | --- | --- | --- | --- | --- | --- | --- | --- | --- | --- | --- | --- | --- |
| BBB size  (buds/m^2^) | < 0.05 | 1690.43 | ± | | 332.71 | 6120.00 | 312.00 | 1100.00 | ( | 704.00, | 1832.00 | ) | |
| Longitude (°) | < 0.05 | 98.59 | ± | | 0.77 | 102.54 | 90.83 | 100.16 | ( | 95.86, | 100.94 | ) | |
| Latitude (°) | > 0.05 | 34.91 | ± | | 0.44 | 38.46 | 30.77 | 34.88 | ( | 33.67, | 36.35 | ) | |
| Altitude (m) | < 0.05 | 3766.84 | ± | | 117.17 | 4742.97 | 3023.00 | 3560.00 | ( | 3281.0, | 4223.91 | ) | |
| AMT (℃) | > 0.05 | 0.52 | ± | | 0.44 | 3.46 | -4.34 | 0.82 | ( | -0.83, | 2.39 | ) | |
| AMP (mm) | > 0.05 | 479.04 | ± | | 37.86 | 861.59 | 209.89 | 462.66 | ( | 323.13, | 602.74 | ) | |
| SAP (mg/kg) | < 0.05 | 8.94 | ± | | 1.44 | 31.57 | 2.68 | 7.24 | ( | 4.46, | 9.48 | ) | |
| SMC (%) | > 0.05 | 16.24 | ± | | 1.92 | 34.87 | 2.01 | 15.62 | ( | 7.42 | 22.67 | ) | |
| Soil pH | > 0.05 | 7.65 | ± | | 0.19 | 9.06 | 6.30 | 7.43 | ( | 6.93, | 8.47 | ) | |
| ST (℃) | > 0.05 | 19.91 | ± | | 1.41 | 34.50 | 10.29 | 18.61 | ( | 13.86, | 23.83 | ) | |
| NH_4_^+^-N (mg/kg) | < 0.05 | 3.16 | ± | | 0.53 | 9.35 | 0.25 | 2.00 | ( | 0.90, | 5.12 | ) | |
| NO_3_^−^-N (mg/kg) | < 0.05 | 12.74 | ± | | 1.69 | 35.30 | 4.77 | 10.61 | ( | 5.59, | 19.07 | ) | |
| TN (%) | > 0.05 | 0.25 | ± | | 0.03 | 0.49 | 0.05 | 0.27 | ( | 0.15, | 0.36 | ) | |
| TC (%) | > 0.05 | 3.42 | ± | | 0.26 | 5.66 | 1.13 | 3.67 | ( | 2.59, | 4.06 | ) | |
| AKP (μmol/d/g) | > 0.05 | 25.43 | ± | | 2.14 | 41.84 | 4.73 | 26.85 | ( | 18.07, | 34.22 | ) | |
| LAP (μmol/d/g) | < 0.05 | 7.61 | ± | | 1.45 | 23.76 | 0.99 | 4.56 | ( | 2.17, | 11.49 | ) | |
| βG (μmol/d/g) | > 0.05 | 43.08 | ± | | 5.24 | 100.80 | 6.50 | 43.58 | ( | 19.26, | 64.94 | ) | |
| PPO (μmol/d/g) | > 0.05 | 20.08 | ± | | 2.72 | 47.25 | 1.39 | 22.50 | ( | 6.04, | 27.58 | ) | |
| ACP (μmol/d/g) | < 0.05 | 18.07 | ± | | 2.32 | 42.58 | 3.77 | 13.34 | ( | 9.50, | 22.73 | ) | |
| BGB (g/m^2^) | < 0.05 | 1435.45 | ± | | 383.62 | 7826.99 | 89.23 | 741.13 | ( | 315.30, | 1787.00 | ) | |
| AGB (g/m^2^) | < 0.05 | 325.26 | ± | | 59.21 | 1118.56 | 69.41 | 198.24 | ( | 156.24, | 370.56 | ) | |
| Margalef | > 0.05 | 1.10 | ± | | 0.07 | 1.73 | 0.58 | 1.08 | ( | 0.73, | 1.38 | ) | |
| Shannon-Wiener | > 0.05 | 0.93 | | ± | 0.09 | 1.76 | 0.35 | 0.78 | ( | 0.61, | 1.41 | ) |  |
| Simpson | < 0.05 | 0.94 | | ± | 0.08 | 1.80 | 0.41 | 0.76 | ( | 0.68, | 1.23 | ) |  |
| Pielou | > 0.05 | 0.67 | | ± | 0.02 | 0.83 | 0.45 | 0.68 | ( | 0.60, | 0.76 | ) |  |

Notes: BBB (belowground bud bank), AMT (annual mean temperature), AMP (annual mean precipitation), SAP (soil available phosphorus content), SMC (soil moisture content), ST (soil temperature), NH_4_^+^-N (soil ammonium nitrogen), NO_3_^−^-N (soil nitrate nitrogen), TN (soil total nitrogen content), TC (soil total carbon content), AKP (soil alkaline phosphatase, EC:3.1.3.1), LAP (soil leucine aminopeptidase, EC:3.4.11.1), βG (soil β-glucosidase, EC:3.2.1.21), PPO (soil polyphenol oxidase, EC:1.14.18.1), ACP (soil acid phosphatase, EC:3.1.3.2), BGB (belowground biomass), AGB (aboveground biomass), Margalef (Margalef index), Shannon-Wiener (Shannon-Wiener index), Simpson (Simpson’s diversity index), Pielou (Pielou’s species evenness index).

# Supplement Figures:


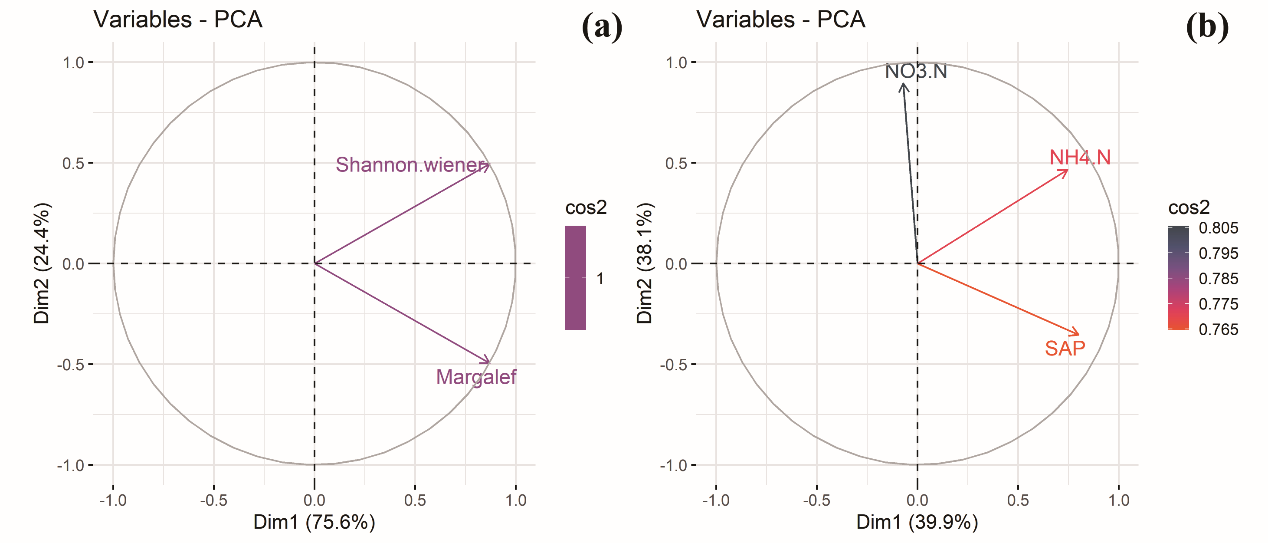


Supplement Fig. 1 PCA was conducted for the selected environment factors (with relative importance >5%, in Fig. 3). PCA can transform multiple factor variables of similar categories into a set of variables, here, converting “Margalef” and “Shannon Wiener” to “Plant Diversity” (a), and converting “NH_4_^+^-N”, “NO_3_^−^-N” and “SAP” to “Soil N-P” (b), subsequently, that will be used to construct SEM.

Notes: Margalef (Margalef index), Shannon.wiener (Shannon-Wiener index), NH4.N (soil ammonium nitrogen), NO3.N (soil nitrate nitrogen), SAP (soil available phosphorus content).
